# Acknowledgement to Reviewers of *Scientia Pharmaceutica* in 2017

**DOI:** 10.3390/scipharm86010004

**Published:** 2018-01-15

**Authors:** Scientia Pharmaceutica Editorial Office

**Affiliations:** MDPI AG, St. Alban-Anlage 66, 4052 Basel, Switzerland

Peer review is an essential part in the publication process, ensuring that *Scientia Pharmaceutica* maintains high quality standards for its published papers. In 2017, a total of 38 papers were published in the journal. Thanks to the cooperation of our reviewers, the median time to first decision was 32.5 days and the median time to publication was 69.5 days. The editors would like to express their sincere gratitude to the following reviewers for their time and dedication in 2017:
Abd El Hakim, YasminaKudelski, AndrzejAchunike, Akah PeterLakritz, JeffreyAgostino, MarkLänger, ReinhardAncuceanu, RobertLanger, ThierryAndersen, Jens RikardtLarraneta, Landa EnekoAnraku, MakotoLászló, KursinszkiAragão, Cícero F. S.Leclercq, JoëlleBanach, MarcinLee, Jae YeolBarth, ThiagoLee, JaehwiBatzias, GeorgiosLeibowitz, AvshalomBedse, GauravLeonidas, Demetres D.Berthelsen, RagnaLiévin-Le Moal, VanessaBirch, JohnLin, Chih-ChienBorg-Karlson, Anna-KarinLodh, NilanjanBracher, FranzLoniewski, IgorBrandão Gomes, Claudia Regina Lopes, Carla M.Brooke, JoanneLopez, Dolores E.Brusaferro, SilvioLores, MartaBucar, FranzManganaris, GeorgeBuchbauer, GerhardMargalef, MariaBurgdorf, JeffreyMartens, JürgenBuscemi, SilvestreMartins, NatáliaCampana, RaffaellaMattingly, JoeyCapasso, RaffaeleMcGregor, Gerard P.Cardia, Maria CristinaMikolajczak, PrzemyslawCarvalho, Ana MariaMing Long, ChiauCascone, SaraMorganti, PierfrancescoCerella, ClaudiaMoyano, EncarnaChauhan, HarshMshvildadze, VakhtangChen, Hsin-ChunMuñoz, Pilar MaríaCheng, Juei-TangNapolioni, ValerioCivelli, OlivierNebot, CarolinaCoimbra, Elaine S.Nigovic, BiljanaCook, PaulNinfali, PaolinoCox, GeorginaNishizawa, DaisukeCzerwinska, MonikaOjo, OpeoluDalmoro, AnnalisaOkafor, FlorenceDas, AninditaOsolodkin, DmitryDawidowicz, Andrzej L.Palavai Sripal, ReddyDe Diego, MartaPalomo, ValleDe La Fuente, Jesus M.Panchal, SunilDe Luca, MichelePapke, Roger L.De Smet, JorenPavic, ValentinaDelorme, VincentPavšelj, NatašaDi Giorgio, CarolePereira Costa, Soraia Katia Di Martino, Piera Plastina, PierluigiDimas, KonstantinosPrior, JoãoDodi, GianinaRadenkovic, MiroslavDonati Zeppa, SabrinaRajić, ZrinkaDotsikas, YannisRatz-Łyko, AnnaDrenan, Ryan M.Rider, CynthiaEspuelas, SocorroRodrigues, FranciscaFernández-Soto, PedroRodriguez-Cabezas, Maria ElenaFest, ThierryRzymowska, JolantaFigueras, AlbertSalaheen, SerajusFlieger, JolantaSanabria-Ríos, David JuliánFyfe, LornaSankaranarayanan, Nehru VijiGaascht, FrancoisSantagati, AndreaGallelli, LucaSchuster, DanielaGan, Ren-YouScorziello, AntonellaGbylik, MałgorzataSessa, AurelioGondova, TatanaShah, DhavalGonzález Martin, JuliàShaikh, Mohd. FarooqGonzález-Maeso, JavierShibata, HiroyukiGreen, BrianSiewert, BiankaGuada, MelissaŠimon, PeterGumieniczek, AnnaSkaltsa, Helen D. Gurney, HowardSmalls, Britanny L.Hajjar, EmilySrinivasan, BharathHamburger, MatthiasStacchiotti, AlessandraHanganu, DanielaStefanowicz-Hajduk, JustynaHautz, TheresaStemer, GunarHernández Núñez, EmanuelStergiopoulos, StellaHooshmand, ShirinStuppner, HermannHroboňová, KatarínaTam, FeliciaIkegaya, NaokiTavano, LorenaInami, KeikoTeixeira, PaulaJäger, WalterTiruveedhula, Veera Venkata Naga Phani BabuJaiswal, RakeshTóth, GergőJakovljevic, MihajloTryndyak, VolodymyrJaradat, NidalUkrainets, Igor V.Jeong, Byoung RyongUngphaiboon, SuwipaJerzsele, ÁkosVecsernyes, MiklosJha, AmitVerpoorte, RobertJohn, ShaliniVlase, GabrielaJuan, M. EmíliaVyazovkin, SergeiKachrimanis, KyriakosYagupsky, PabloKamel, KarolYamada, HiroyukiKao, Erl-ShyhYamamoto, NorioKetkar, AmitYang, JiaoKim, BohyungYoh, TakekumaKinoshita, TakayoshiZamfir, MedanaKipper, KarinZbytovska, JarmilaKrenn, LiselotteZhang, HuKrystof, VladimirZia, Muhammad

